# Supplementary figures and images for: The role of ducks in detecting Highly Pathogenic Avian Influenza in small-scale backyard poultry farms
Source: PLoS Comput Biol. 2026 Jan 9;22(1):e1013357. doi: 10.1371/journal.pcbi.1013357 (PMC12810905; doi:10.1371/journal.pcbi.1013357)

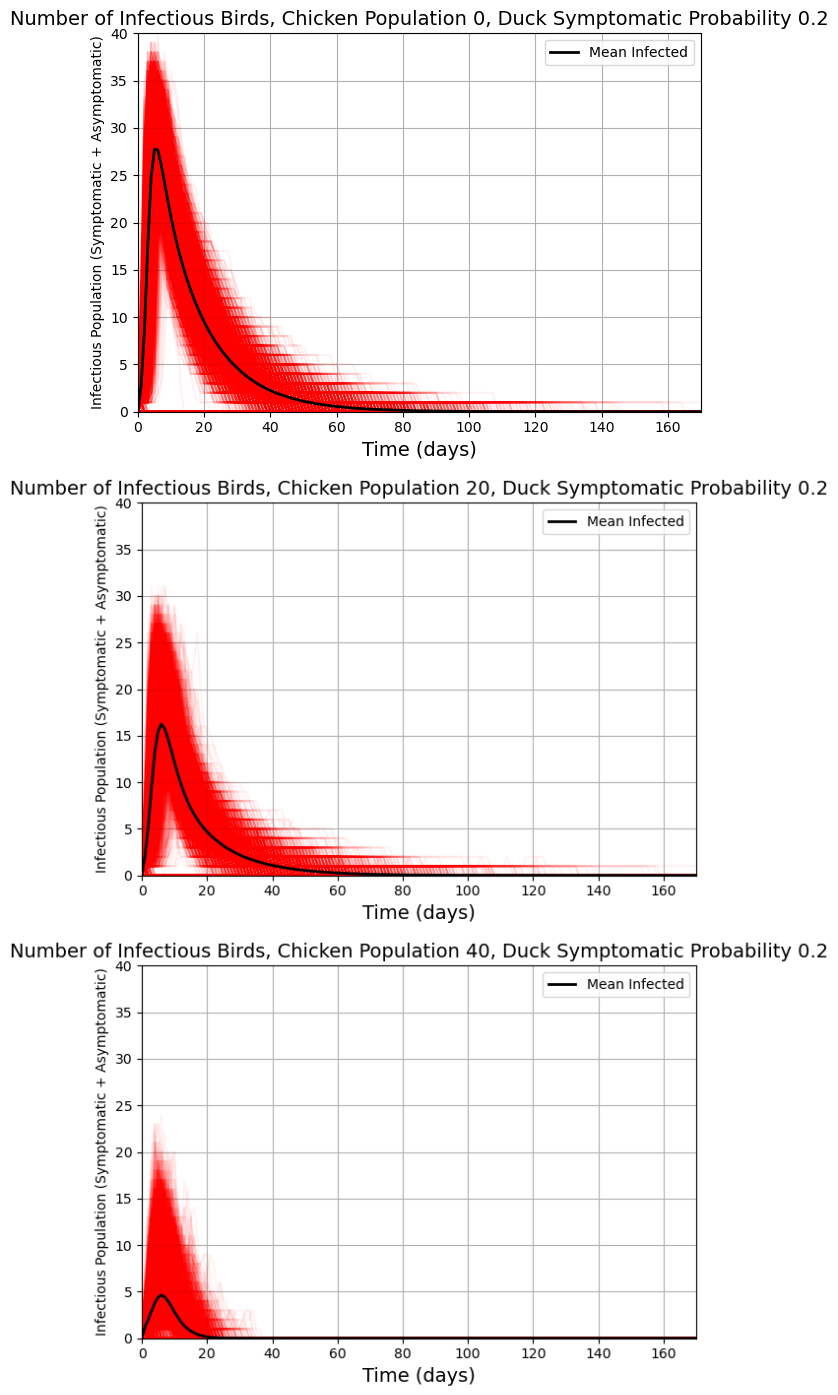

Supplement: S1 Fig — Number of infectious birds over time, for fixed pd = 0.2. Red trajectories shows the time series directly, and the black trajectory is plotted by calculating the average across all time series on each day. Top: all 40 birds are ducks, middle: 20 chickens and 20 ducks, bottom: all 40 birds are chickens. (TIFF) [file pcbi.1013357.s001.tiff]

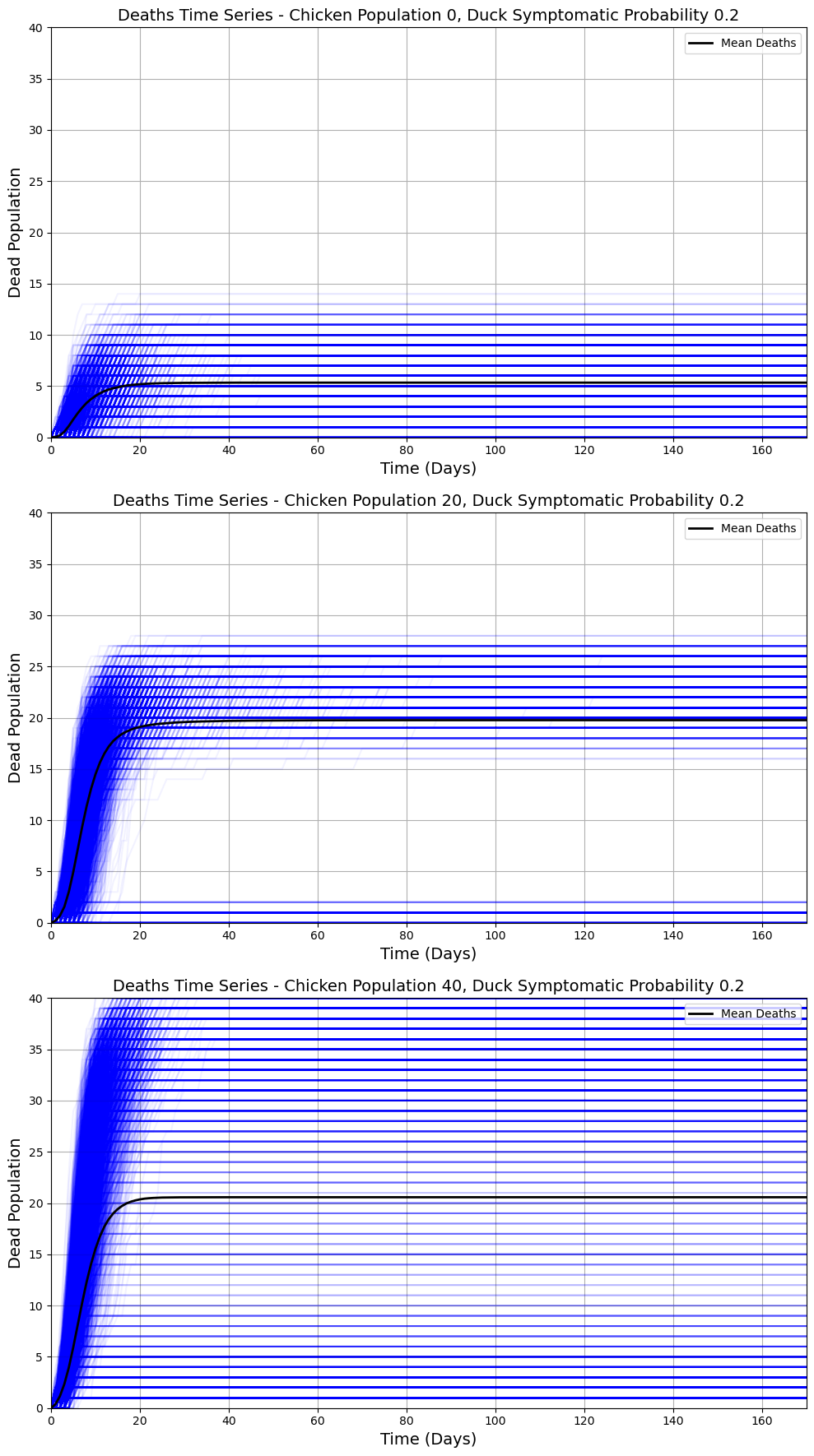

Supplement: S2 Fig — Number of dead birds over time, for fixed pd = 0.2. Blue trajectories shows the time series directly, and the black trajectory is plotted by calculating the average across all time series on each day. Top: all 40 birds are ducks, middle: 20 chickens and 20 ducks, bottom: all 40 birds are chickens. (TIFF) [file pcbi.1013357.s002.tiff]

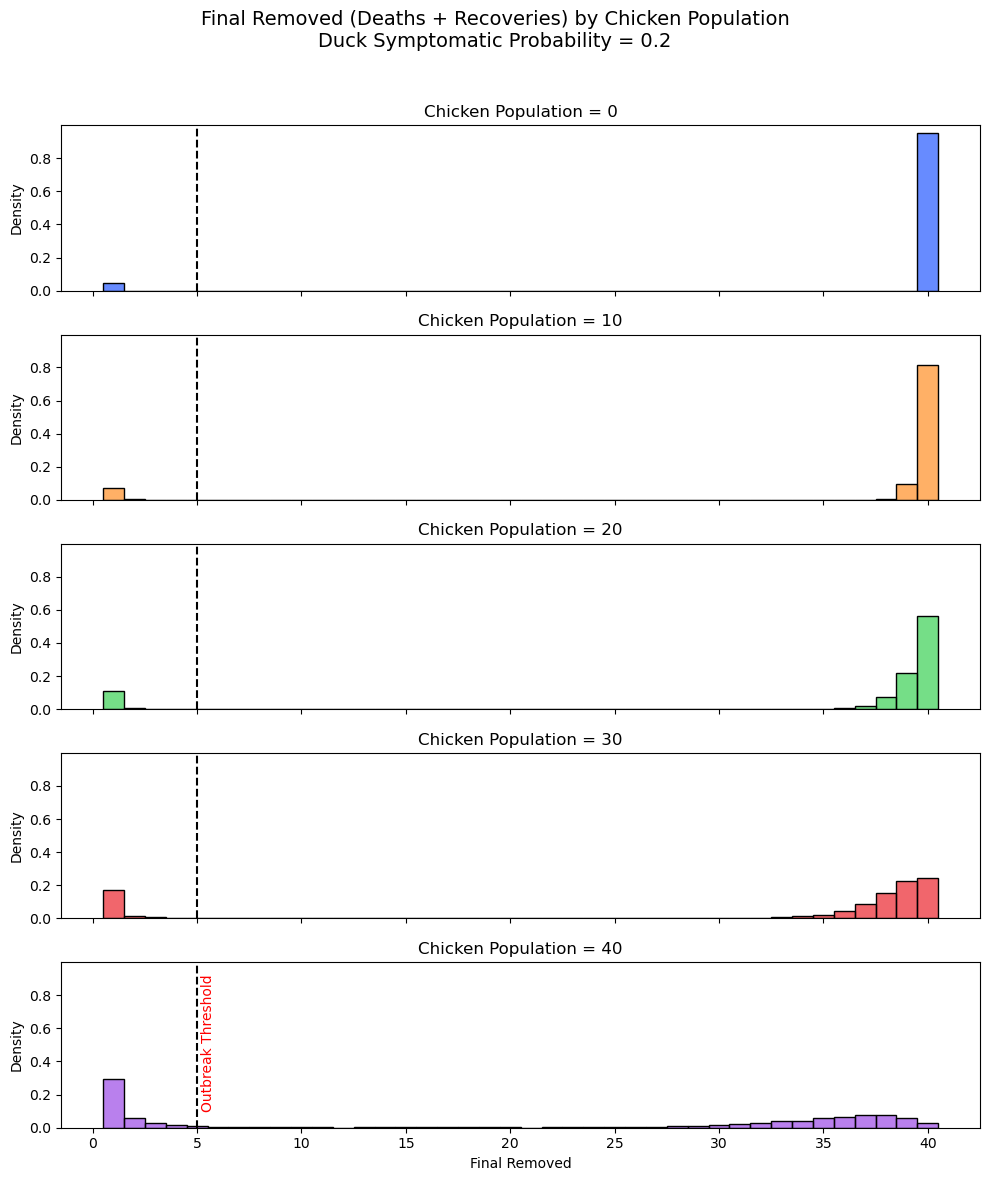

Supplement: S3 Fig — Histogram of distribution of final removed numbers (dead or recovered) with varied number of chickens in flocks of size 40. Duck symptomatic probability set to 0.2. The vertical dashed line indicates time series that are considered an outbreak (To the right of the dashed line). (TIFF) [file pcbi.1013357.s003.tiff]

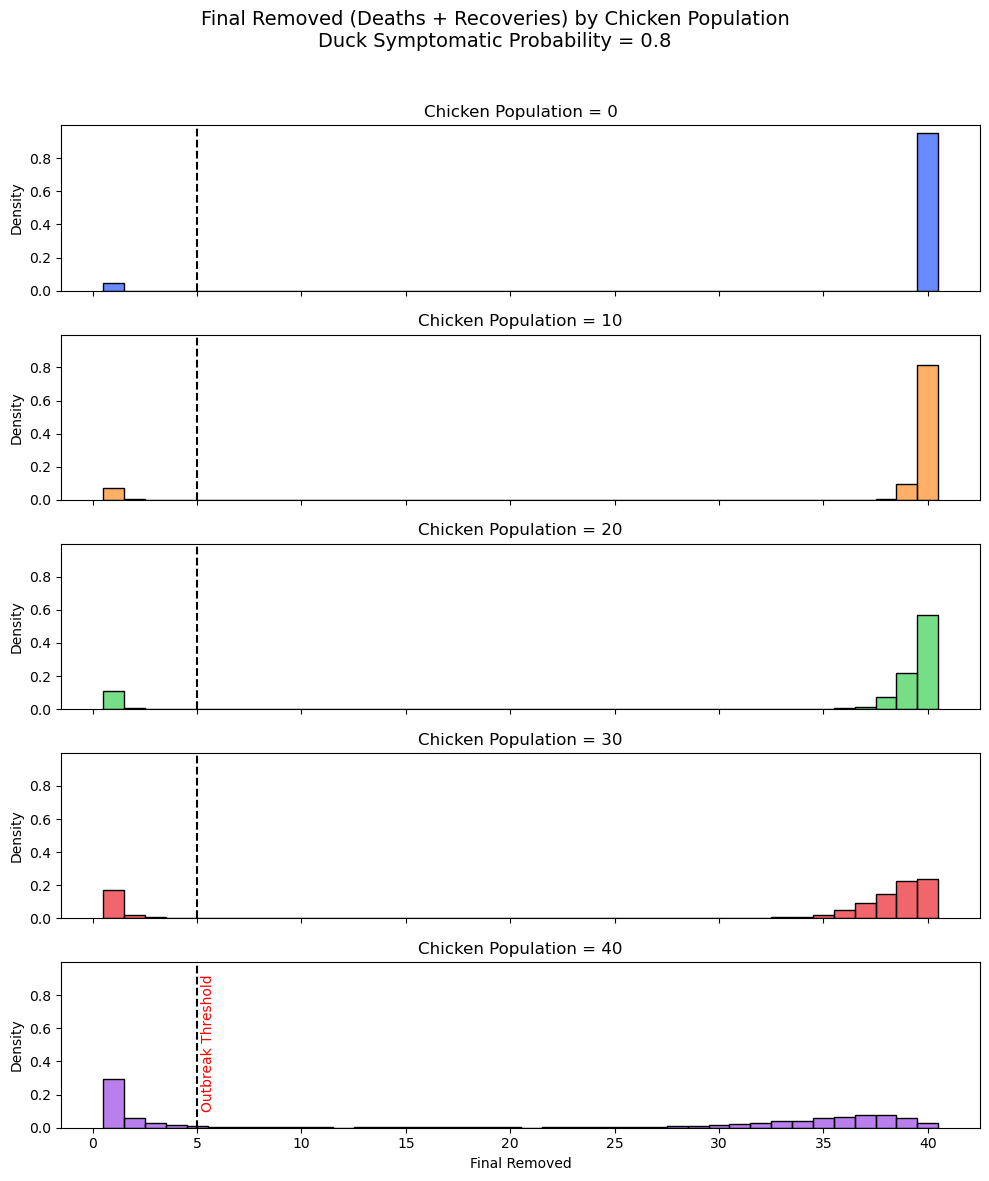

Supplement: S4 Fig — Histogram of distribution of final removed numbers (dead or recovered) with varied number of chickens in flocks of size 40. Duck symptomatic probability set to 0.8. The vertical dashed line indicates time series that are considered an outbreak (To the right of the dashed line). (TIFF) [file pcbi.1013357.s004.tiff]

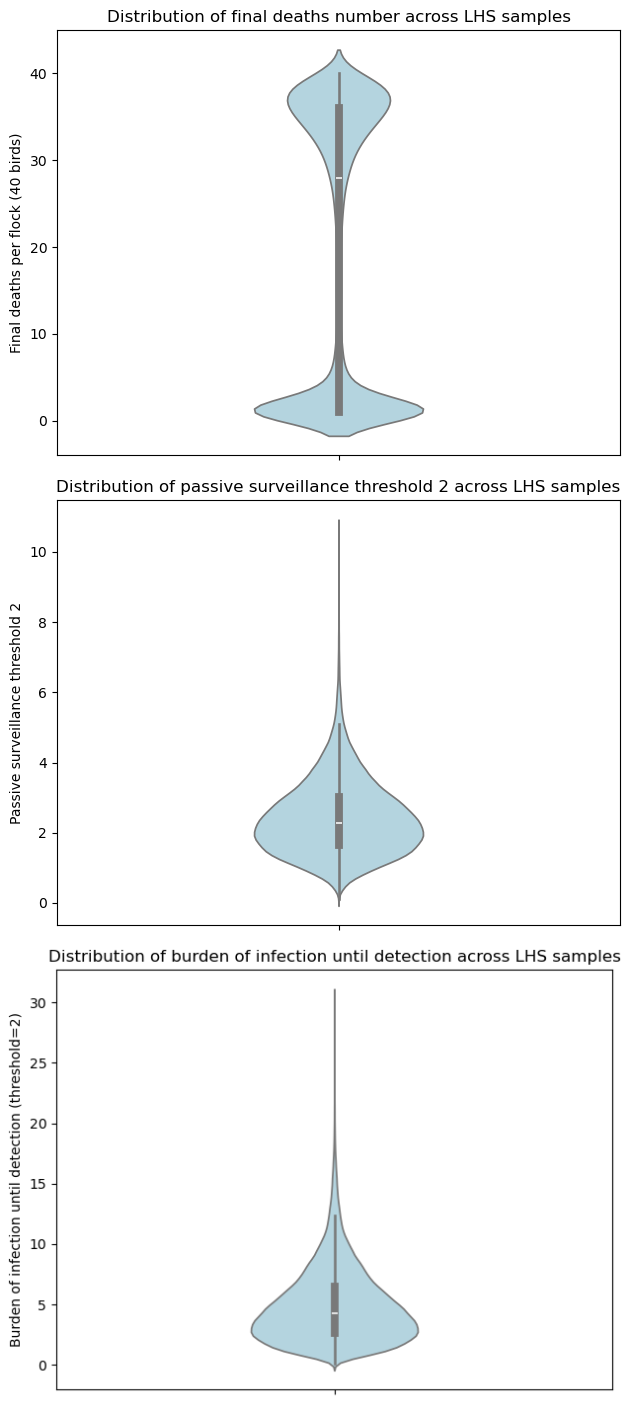

Supplement: S5 Fig — Violin plot for the distribution of final deaths, detection time, and burden of infections among all LHS simulations (all-chicken flocks). (TIFF) [file pcbi.1013357.s005.tiff]

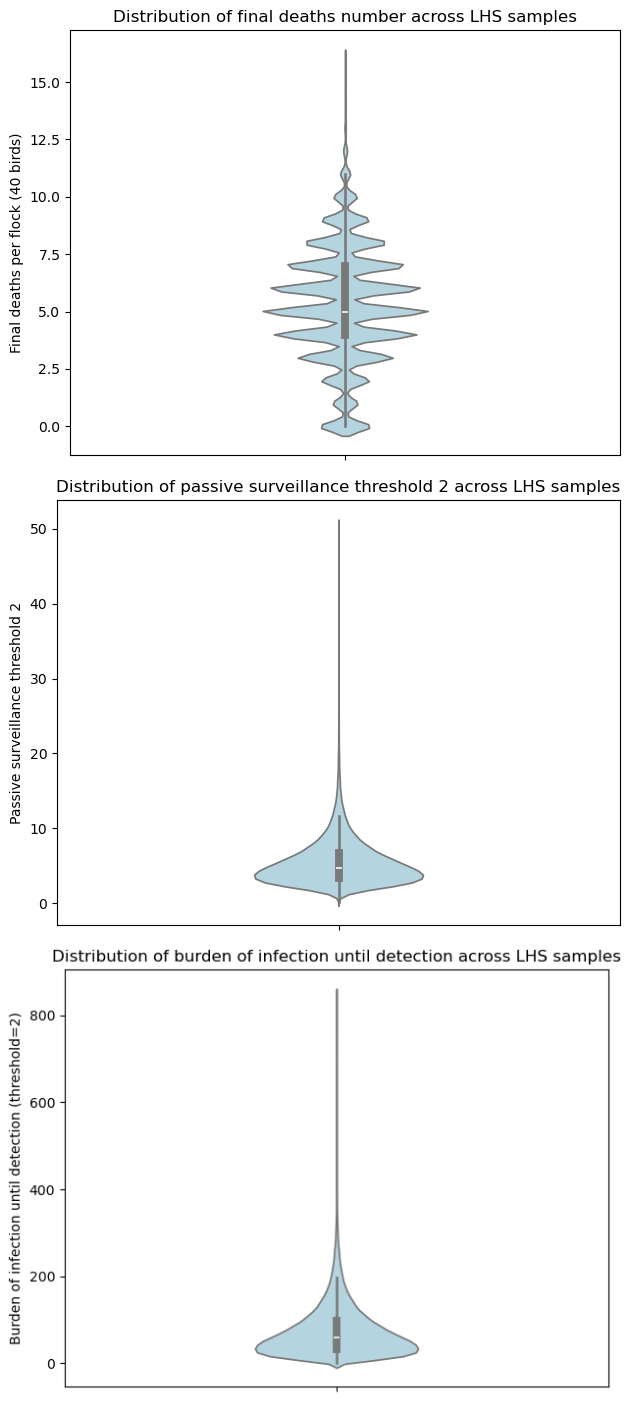

Supplement: S6 Fig — Violin plot for the distribution of final deaths, detection time, and burden of infections among all LHS simulations (all-duck flocks). (TIFF) [file pcbi.1013357.s006.tiff]

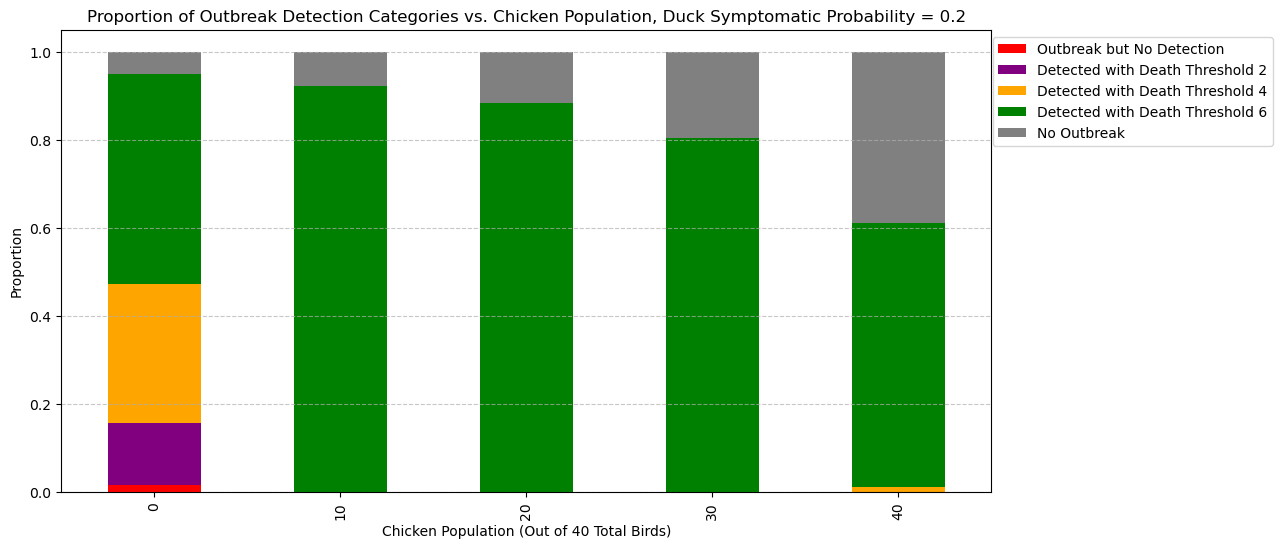

Supplement: S7 Fig — Bar plot showing the probability of a simulation run resulting in an outbreak, and whether it would be detected with death thresholds 6, 4, or 2. This is fixed with pd = 0.2. For pd = 0.8 all outbreaks can be detected with the highest death threshold. (TIFF) [file pcbi.1013357.s007.tiff]

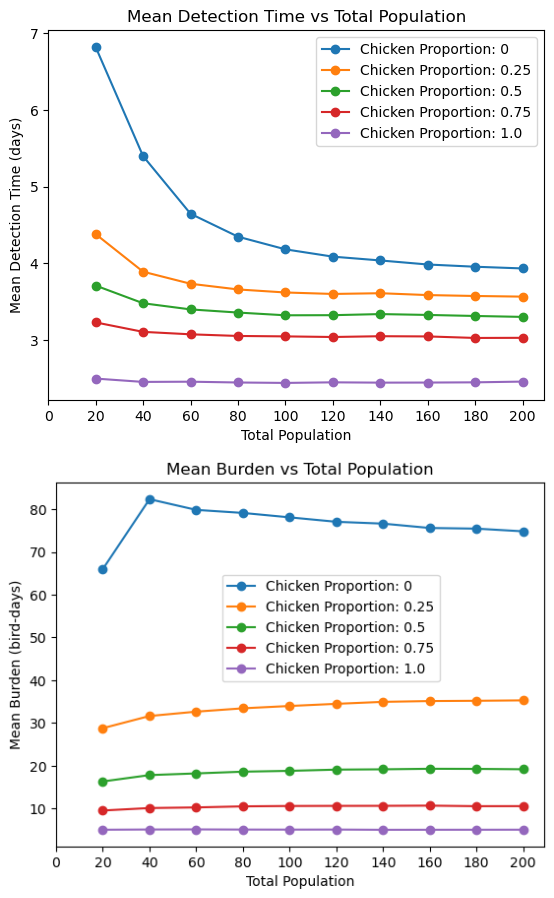

Supplement: S8 Fig — The mean detection time (top) and burden of infection (bottom) until detection for different total flock sizes and the proportion of chickens in the flock. Other parameters, including pd = 0.2, were fixed. (TIFF) [file pcbi.1013357.s008.tiff]

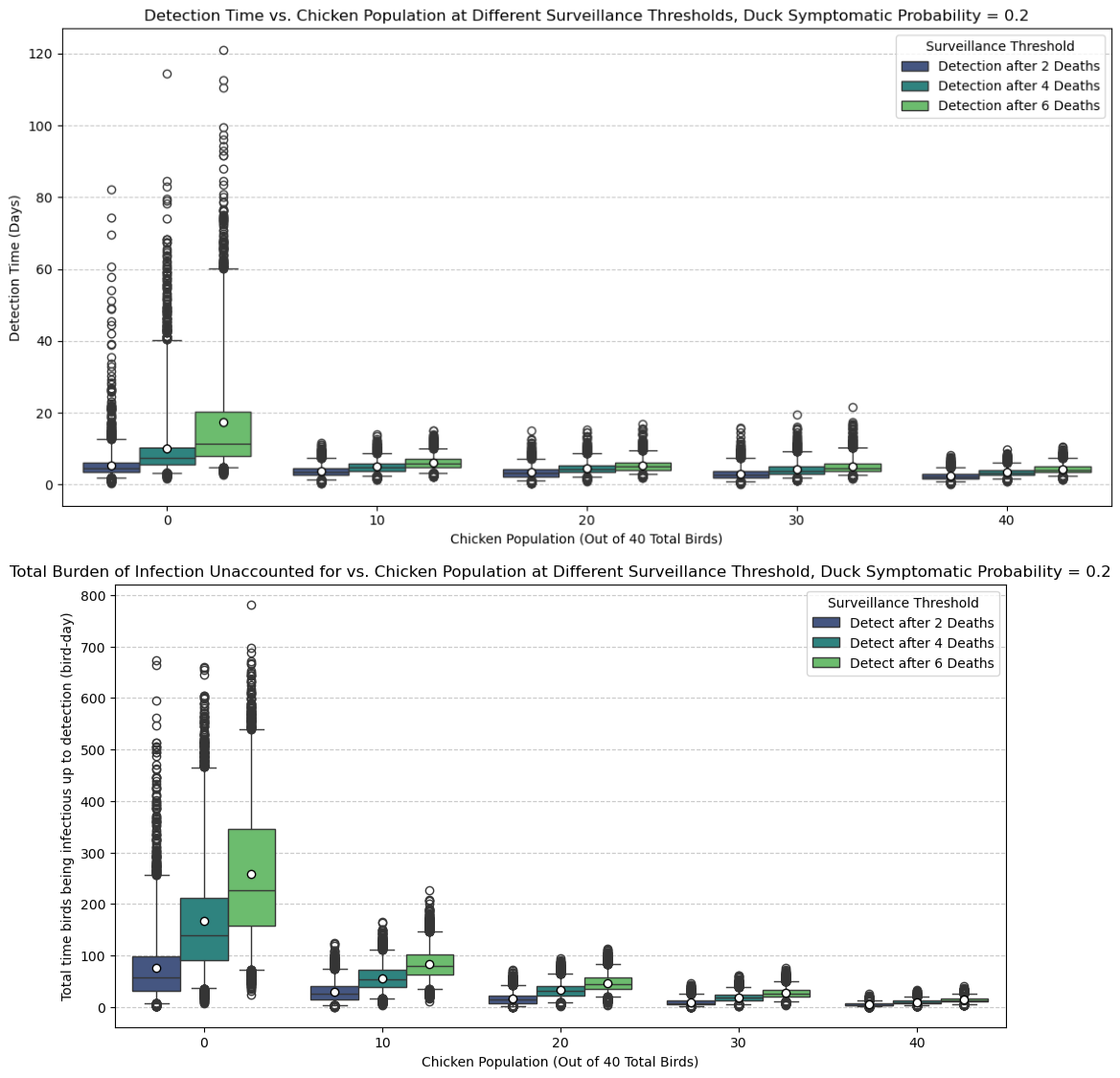

Supplement: S9 Fig — The detection time and burden of infection for the natural mortality rate 10−3 (approximately 30% yearly mortality without HPAI). This shows that the inclusion of natural mortality will not have any qualitative impact on our results. (TIFF) [file pcbi.1013357.s009.tiff]
