# Supplementary material for: The role of ducks in detecting Highly Pathogenic Avian Influenza in small-scale backyard poultry farms
Source: PLoS Comput Biol. 2026 Jan 9;22(1):e1013357. doi: 10.1371/journal.pcbi.1013357 (PMC12810905; doi:10.1371/journal.pcbi.1013357)
Supplement: Attachment — Submitted filename: Response to reviewers.pdf [file pcbi.1013357.s011.pdf]

Dear editors,

Thank you giving us the opportunity to revise our manuscript. We have responded to the reviewers' suggestions below, with appropriate changes made in our manuscripts. In each case we have provided a detailed response to the reviewer comments.

Overall, the reviewers' comments have been very useful and improved the clarity and robustness of our manuscript. However, we strongly disagree with several of the points raised by reviewer 2, particularly in reference to the validity of the mixing assumption and the fact that it is based upon a standard SIR modelling approach with no "significant methodological innovation". We disagree with the reviewer that this is a requirement for PLOS Comp Bio given our experience of previous publications in the journal. We have greatly extended our sensitivity analysis in response to comments by reviewer 2 and believe that this has strengthened the paper, but we have also rebutted several of the points made by reviewer 2 because we simply disagree with them. In this case we hope that the editorial team will be able to evaluate our response to reviewer 2 given that we don't believe that this reviewer will look favourably upon our rebuttal to their comments.

Sincerely,

Steven X Wu, Chris N Davis, Mark Arnold, Mike J Tildesley

Reviewer #1:

In this manuscript, Wu et al. presents a well-designed simulation study examining the transmission dynamics of Highly Pathogenic Avian Influenza (HPAI) H5N1 within small-scale, mixed-species poultry flocks, with particular attention to backyard farming systems. The authors develop a computational model that simulates disease spread, incorporating factors such as species composition, chicken-to-duck ratios, mortality-based detection timing, and cumulative infectious burden prior to detection. This approach captures important complexities often overlooked in models designed for large, single-species commercial farms.

Overall, the manuscript is well written and addresses a timely and relevant topic. However, several aspects would benefit from further clarification and elaboration. Please see my comments below:

Major comments:

Abstract:

1. The authors could consider mentioning in the abstract that the authors have developed a computational model to simulate the spread of HPAI H5N1.

We agree with this suggestion and have added, "computational, mechanistic model" in the abstract to clarify this.

2. Including the GitHub link in the abstract would improve accessibility and encourage use of the model and data.

Whilst we agree with the need to improve accessibility of model code, we don't agree that adding a GitHub link in the abstract is appropriate, as the abstract should focus upon the scientific content that we are presenting. However, we note that there will be a code availability section in the paper should it be published in this journal and additionally we have edited the methods section to provide a link to the GitHub repository. We believe that this is the most appropriate place for this, though if the editor disagrees, we are willing to make the change suggested by the reviewer.

Introduction:

3. Add a brief (2–3 sentence) overview of why HPAI H5N1 matters globally and why backyard flocks are a unique concern.

We have already emphasised this point in the first paragraph of the introduction, but to strengthen this we have added additional context for other countries that we feel helps to consolidate our point. We have also added the following (with a reference to a survey study) to justify the significance of studying backyard flocks:

"Between October 2021 and April 2022, approximately a quarter of all HPAI-infected premises in the UK are consider 'smallholding' or 'backyard', demonstrating the significance of studying these types of flocks."

"In Southeast Asian countries such as Indonesia, backyard farms were found to have a higher contact rate than other types of flocks and were most at risk for transmissions of HPAI..."

4. Highlight novelty by emphasizing what makes your study different from existing research.

We agree that it is important to highlight the novelty of the work and we have expanded our discussion in response to this and comments from other reviews to emphasise what we believe is particularly novel about our research.

5. Briefly outline how the findings could inform public health or agricultural policy.

We have now expanded the penultimate paragraph of the discussion, emphasising that future policies should balance between reducing mortality and limiting transmission, and raise awareness of H5N1 signs among smallholder farmers. This helps to demonstrate how our findings can inform public health and agricultural policy.

6. Discuss limitation by adding a short note on what the model does not capture (e.g., environmental factors, human intervention) adds credibility.

We agree with this point and have expanded the discussion to focus on limitations of the work, including mentioning different breed of birds, age-related mortality, and non-homogeneous contact patterns.

Methods:

7. The simulation-based approach is strong, with thoughtful use of the Gillespie algorithm and behavioral detection thresholds. However, the assumed flock size of 40 birds may limit generalizability. Absolute mortality thresholds (e.g., 2–6 birds) represent a large proportion of the flock and may not scale well to larger operations. Smaller flocks are also more susceptible to stochastic effects. To improve applicability, I suggest including simulations with larger flock sizes or a sensitivity analysis to assess how outcomes vary with population size.

We agree that the results here may be dependent upon flock size and therefore we have added plots (Supplementary figure S8) that compares detection time/burden of infection for different total flock sizes (from 20 to 200, with 20 increments), with 0%, 25%, 50%, 75%, 100% of them being chickens, fixing $p_d = 0.2$. We also mention that our original choice of 40 birds may have overestimated detection time/burden of infections, but duck-only flocks remain highest risk.

8. The authors have acknowledged the key model assumptions such as homogeneous mixing and exclusion of natural mortality. It would be helpful to more clearly discuss how these assumptions might influence transmission dynamics and detection timing.

We agree with the reviewer that it is important to address this limitation, and we have expanded the discussion to acknowledge this point (see response to point 6 above).

9. The authors have provided the GitHub link for data and code. To improve usability,

they should consider adding a README file with clear instructions on how to run and interpret the model.

We have included a README file that gave instructions on how to run the files. More detailed descriptions are within each .ipynb file.

Results:

10. The differences between duck-only and chicken-only flocks are interesting. To strengthen the interpretation, the authors should more clearly explain how biological traits such as longer infectious periods or lower mortality in ducks relate to the simulation results.

We agree with the reviewer and have added the following sentence in the discussion to address this point: "The simulation results match expectations, as ducks have a longer infectious period and lower mortality rate, which causes prolonged infection time and more difficulty in detection."

11. The analysis of detection time at various death thresholds is valuable. To improve its practical relevance, it would help to discuss how these thresholds reflect real-world surveillance practices, particularly in smallholder and commercial settings.

We use multiple death thresholds to represent a range of farmer behaviours. In the discussion, we acknowledge this difference, and we have now added a recommendation based on passive surveillance.

"In general, we also recommend raising awareness of H5N1 signs and symptoms among smallholders, particularly hobby farmers, to reduce the detection time and subsequently the burden of infections using the passive surveillance approach."

12. Using bird-days to measure infectious burden is appropriate. To highlight its epidemiological importance, the authors could more clearly explain how a higher burden might increase the risk of transmission to nearby flocks or humans.

We have now explained this explicitly in the discussion with the following sentence:

"We also found that ducks tend to contribute to a higher unnoticed burden of infection, which implies that more birds stay infectious for a longer time, potentially resulting in a higher risk of between-flock transmissions through direct or indirect contact."

13. The decision to analyze only simulations with detected outbreaks may underestimate the true burden of infection, particularly in duck-only flocks. A brief

discussion or sensitivity analysis of undetected outbreaks would provide a more complete picture.

We thank the reviewer for the suggestions and have included a bar plot in the supporting information to show the proportion of undetected outbreaks. Undetected outbreaks will be most relevant in duck-only flocks for the case of small p_d. If we included undetected outbreaks, it would only lead to a higher value of detection time/burden of infection, which reinforces our conclusion.

Discussion:

14. The authors acknowledge variability in farmer behavior, which is commendable. However, the discussion could be strengthened by referencing more region-specific behavioral studies or proposing how behavioral heterogeneity could be modeled in future work.

We agree with the reviewer on the importance of farmer behaviour. Farmer behaviour can potentially have a substantial influence on dynamics both within and between premises and the senior author on this paper has done some recent work in this area for foot-and-mouth disease, demonstrating the impact of heterogeneous responses to outbreaks on large scale dynamics. We therefore cite this work (Hill et al. (2023) "Incorporating heterogeneity in farmer disease control behaviour into a livestock disease transmission model") and discuss the impact of behaviour in the discussion section of the paper.

15. The discussion mentions important assumptions, such as homogeneous mixing and the exclusion of natural mortality. However, their impact on the results should be explored in more depth. For instance, how could spatial separation or age-related mortality affect detection patterns?

As in our response to point 6, we have expanded upon this in the discussion of the paper. In particular, we have added an explanation on how these factors might affect detections.

16. The author could consider presenting the balance between reducing mortality and limiting transmission as a multi-objective optimization problem to strengthen the study's computational relevance.

We agree this would be an interesting analysis and added a few sentences on this to the discussion.

“A future direction in researching H5N1 transmission is to optimise intervention strategies by balancing reducing mortality and limiting transmission. In general, we also recommend raising awareness of H5N1 signs and symptoms among smallholders, particularly hobby farmers, to reduce the detection time and subsequently the burden of infections using the passive surveillance approach.”

17. The proposed future work is appropriate but could be expanded to include agent-based models, spatial simulations, or integration with real-world surveillance data. Exploring these directions would broaden the study’s scope and enhance its impact.

We agree with the reviewer and have added these methods to our proposed future work in the final paragraph.

“The parameter estimation may be integrated with real-time surveillance data and can be used on extended models such as spatial simulations or agent-based models.”

Reviewer #2:

The paper addresses an interesting research question related to modeling influenza transmission across different avian species. The manuscript is generally well written with a clear presentation of the methodology. However, after careful review, I have concerns regarding the significance of the problem, the novelty of the methodology, and the design of the numerical experiments.

Regarding the problem setup, the authors emphasize that interspecies transmission in avian populations has not been investigated. The focus of the article is on small backyard settings where there is limited or no data available to validate the parameter values used in the dynamical system models.

This raises concerns about the practical relevance and credibility of the simulation results. While small backyard scenarios are underexplored, higher-density industrial settings may be of greater interest from both scientific and public health perspectives.

Additionally, data from such settings may be available to support parameter estimation and model validation.

I think incorporating these would significantly enhance the attractiveness and trustworthiness of the study in real-world applications, or the authors should explain more clearly why they chose this setting given the limited data resources.

While industrial farms are of greater significance in terms of infection potential in a UK setting, there is already an abundance of studies on those farms. Our argument is that backyard flocks should not be ignored, despite the fact that current literature seems to focus on large-scale farms. Additionally, backyard farms have played a substantive role in the spread and persistence of HPAI for many years in South East Asia, not least because of their potential for interaction with wild avian species.

We have added a reference to show the significance of backyard flocks in the UK, as an example:

"Between October 2021 and April 2022, approximately a quarter of all HPAI-infected premises in the UK are consider 'smallholding' or 'backyard', demonstrating the significance of studying these types of flocks."

We also referenced a paper on backyard flocks in Indonesia:

"In Southeast Asian countries such as Indonesia, backyard farms were found to have a higher contact rate than other types of flocks and were most at risk for transmissions of HPAI…"

This demonstrates that backyard flocks have a significant impact on H5N1 transmissions in SEA, and potentially the UK too. Hence, we argue that our study is highly relevant from a public health perspective.

Regarding the methodology, the main novelty of the paper appears to lie in modeling transmission under a mixed-species structure. However, this novelty is weakened by the use of a simplified, uniform distribution of species.

Since this mixing structure is central to the contribution of the paper (putting aside the backyard setting), I suggest that the authors explore other mixing assumptions that reflect different contact probabilities or densities between species based on real-world scenarios. This would make the methodological contribution more convincing.

In this paper we aim to present an illustrative example of two different species of birds mixing in a backyard setting, rather than a full characterisation of all potential contact behaviours in birds. As the reviewer previously states, little data are available on contact probabilities of different bird species and data that exist are highly context specific. It would be unclear how to parameterise a detailed contact structure without introducing additional specific assumptions, which could be potentially misleading. Therefore, we have chosen a homogeneous mixing structure to keep the methodology transparent and minimise the number of assumptions. We have highlighted this as a limitation in the discussion and suggested some alternative possible assumptions, if more data were available. Since we have also conducted sensitivity analysis on key

parameters as the reviewer suggested below, we believe our model represents a novel approach to explore the risk associated with HPAI outbreaks in backyard flocks.

I do not see other significant methodological innovations that distinguish the manuscript, as the model is based on traditional compartmental formulations and simulations with parameter values drawn from previous references.

I think for a journal like PCB, some advances in computational methods should be proposed, or at least the authors should adopt other advanced existing computational frameworks to draw conclusions instead of relying solely on traditional methods.

We strongly disagree with the reviewer here and believe that they have misunderstood the remit of PLOS Computational Biology. PLOS Comp Bio regularly publishes epidemiological modelling papers of this nature and in fact several papers by these authors have been published in this journal. These papers have not focused upon advances in computational methods per se, but rather the use of computational methodologies to make advances in understanding real world systems. We defer to the editor in this point but given the vast body of previously published epidemiological modelling papers in PLOS Comp Bio, we believe that the reviewer is mistaken on this point.

For the numerical experiments, I have concerns about the validity of the results, as parameter values are taken from previous references that may not be fully suitable for backyard settings. Given that no data are available, the resulting conclusions have limited support from real-world scenarios.

We disagree with the reviewer – this is a standard approach in epidemiological modelling, whereby if data are not available to ascertain all parameter values, then parameters are typically taken from the literature but sensitivity to precise parameter values is carried out. Our model parameter values have been selected based upon suitable values in the literature wherever possible (and appropriate references have been cited). Indeed, our chicken-to-chicken transmission rate parameter is taken from a source specific to the 'backyard chickens and fighting cocks' setting in Thailand (Tiensin et al., 2007). As stated below, we have also extended our sensitivity analysis to ensure that we robustly explore the impact of our assumptions on the model outcomes. This is a widely used approach in epidemiological modelling.

I therefore suggest that the authors explore potential datasets to help validate the research findings, or change the problem settings to ones for which data validation is possible.

We acknowledge that there is a lack of real-world data and this poses a challenge for validating results. However, the sparsity of data and therefore lack of focus on backyard flocks in the literature is also what motivates our manuscript. We use the best available parameter estimates to consider this under-explored setting and then consider the sensitivity of these parameters. We feel that changing the problem setting defeats the purpose of our study, which is to understand the infection dynamics in these mixed-species backyard flocks.

The design of the sensitivity analysis for some model parameters also confuses me, as only a few values are considered, which may be too simplistic for a highly nonlinear SEIR model with mixed densities assumed.

I suggest the authors switch to a continuous distribution for the sensitivity analysis instead of testing only a few points. Consider using Latin Hypercube Sampling if no data are available.

The purpose of sensitivity analysis should be to compare and identify parameters from the full set that are of interest. For example, people may be most interested in the transmission rate and asymptomatic probability, which are more sensitive and therefore more crucial for controlling the spread.

Based on this, a full comparison of sensitivities for all parameters is needed.

As in our response to other reviewers, we have extensively expanded our sensitivity analysis to include Latin Hypercube Sampling and also to investigate the impact of epidemiological parameter values upon our model outcomes. Additional figures (Figure 9 and 10, Supplementary figure 5 and 6) have been included in the results section. As can be seen from these additional figures, most parameters seemed to not have a significant qualitative impact on the result, with |rho| < 0.3. A small number of parameters (duck-to-duck transmission rate, kappa) have a moderate impact 0.3 < |rho| <0.4 on the detection time in duck-only flocks. These findings suggest that our main conclusions are robust to variation in most epidemiological parameters, with only limited sensitivity observed for specific parameters in particular flock compositions.

Reviewer #3:

Overall comment:

This study evaluates the implications of including ducks in mixed-species, small-scale backyard flocks for the transmission and detection of HPAI H5N1 virus. As ducks are

relatively less susceptible to this virus, the findings are largely expected. Nevertheless, the authors present the epidemiological impacts in a systematic and informative way. I believe, however, that additional work is needed to be done to strengthen the manuscript for publication in this journal. My main comments relate to: (1) background mortality, (2) model assumptions, and (3) sensitivity analyses.

Specific comments:

Line 6: This example seems too specific, especially given that the journal targets an international readership. Nationwide H5N1 outbreaks have also occurred in other European and non-European countries.

We agree and have removed this example.

Line 11: It may also be relevant to note that outbreaks were reported in cat shelters and mink farms, where close human-to-cat contact is common, for example, in both South Korea and Europe during comparable periods.

We have added the suggested examples to demonstrate the variety of mammal infections, as well as the global significance of H5N1.

Line 44: It would be great to add contextual background on small-scale backyard flocks: indicate where these production systems are prevalent, and explain how this context affects the usefulness of your results.

We added the following (with a reference to a survey study) to justify the significance of studying backyard flocks:

“Between October 2021 and April 2022, approximately a quarter of all HPAI-infected premises in the UK are consider ‘smallholding’ or ‘backyard’, demonstrating the significance of studying these types of flocks.”

“In Southeast Asian countries such as Indonesia, backyard farms were found to have a higher contact rate than other types of flocks and were most at risk for transmissions of HPAI…”

Line 75: Given that you are considering ‘mixed species’ and ‘backyard’ flocks, how reasonable these assumptions are? On backyard farms, birds have relatively more freedom to move around compared to those on commercial farms, hence they may not interact homogeneously. Moreover, you have two different species in the same flock. I think these conditions might have some implications for the homogenous and density-dependent assumptions?

We believe that density-dependent models are more appropriate for small flocks. Unlike in large-scale farms, where each bird is more likely to have a fixed number of contacts regardless of population increase, in a small backyard the population size directly impacts the contact rate of birds. The homogenous assumption can of course

be challenged, as we addressed in the discussion (e.g. chickens and ducks kept separately, etc.). We also use a literature parameter for chicken-to-chicken transmission rate that is specific to backyard chickens (Tiensin et al. 2007).

Line 96: I appreciate that the authors acknowledged the limitations of the available transmission parameters. One additional point I would like to raise is the heterogeneity that exists among chicken breeds, which may be particularly relevant given that the study population includes settings where indigenous breeds are commonly raised alongside ducks. For example, Sonali and Desi chickens exhibit different susceptibility to infection compared with commercial broiler breeds in Bangladesh where mixed-species flocks are common (e.g. https://wwwnc.cdc.gov/eid/article/24/12/18-0879_article). If the aim of the study is to assess the impact of birds with different infection courses, it may be helpful to clearly define "chickens" as commercial broiler breeds at the beginning of the manuscript. I believe commercial broiler chickens are what you meant based on my reading of the following paragraph.

We have now defined chickens to be layers in the method section, since they are the most likely in a backyard setting – and addressed the potential limitation of not exploring different types of birds in the discussion.

"… Additionally, the breeds and types of birds (e.g. broiler or layer) may also influence key parameters, which we did not account for in our simulations…"

Line 146: It would be helpful to define these thresholds as 'background mortality' and include them in Table 1, with appropriate references. In fact, I also wonder why background mortality was not incorporated directly into the model. Moreover, outbreak detection depends not only on background mortality but also on farmers' sensitivity to observed deaths. Stochastic behaviour of these elements would impact detection, along with the role of ducks in your model? The current approach may oversimplify outbreak detection dynamics. I encourage the authors to incorporate these aspects into the model or, at minimum, discuss them in greater detail in the discussion.

Line 173: What would be the impact of background mortality and its difference between chickens and ducks (if present)? I would expect the effect to be negligible, given the relatively small flock sizes and short outbreak periods. However, it would have been helpful if the authors had addressed this in their model or at least showed that they had considered and justified this assumption.

The reason to exclude background mortality (mostly with age-related mortality in mind) is because smallholders tend to identify traits like age in their birds better, as such they are more likely to identify them as such, not H5N1-induced. Furthermore, we are only considering a short time period of approximately three months, so we believe it is reasonable to ignore natural mortality. Otherwise, it is difficult to justify a suitable parameter choice for natural mortality. Of course, this is an assumption, so we

addressed it in the discussion, see third paragraph. The critique on farmer sensitivity was valid, and we also discussed in the same paragraph.

Line 245: And there contact patterns might not be homogeneous...

We agree and therefore we have reiterated and addressed the potential impact on our results in the discussion.

Table 1: Please change Sensitivity and Derived into Assumed. You could explain different values of *k* and *p_d* values were tried as part of sensitivity analysis.

We have updated the table in response. We have also changed the method of sensitivity analysis, see below.

Line 134: What about conducting sensitivity analyses on transmission parameters derived from the literature? Given the limited number of studies, particularly those informing transmission rates, I think sensitivity analyses of these parameters are important. For instance, would the same trends hold under higher or lower transmission rates? Currently, sensitivity analyses were done assuming these parameter values are solid.

We have now used Latin Hypercube Sampling to conduct sensitivity analysis for most parameters. The results are updated accordingly. This included the analysis of transmission parameters.

Reviewer #4:

The paper by Wu et al. The role of ducks in detecting Highly Pathogenic Avian Influenza in small-scale backyard poultry farms, develops a SEIRD model and uses stochastic simulation to study avian influenza transmission between ducks and chickens in small mixed flocks. The parameters are drawn from literatures, and the simulations appear plausible. The work investigates the role of ducks in avian flu transmission in small population, which is interesting. And the authors have acknowledged the limitation of their work.

I have a few concerns:
1. In the text, beta^*_dc=beta_cc/kappa, however, it is inconsistent with kappa*beta_cc in Table 1. Please double check this and the corresponding simulation.

We thank the reviewer for noting the typo in Table 1.

2. In detection time section, the work states that ‘due to higher mortality rate for ducks’, which is misleading as they specifically mentioned that chicken has nearly 100% death rate while the duck actually has a lower case-fatality rate. I think this statement requires clarification with biological evidence.

The higher mortality rate refers to the numerical case in the sensitivity analysis when the probability of showing symptoms is high, compared to the other choices. The wording has now been changed to avoid confusion.

“… However, if the duck symptomatic probability is high, then the overall mortality rate will also increase, and thus the detection time is significantly lowered…”
