# Supplementary material for: The role of ducks in detecting Highly Pathogenic Avian Influenza in small-scale backyard poultry farms
Source: PLoS Comput Biol. 2026 Jan 9;22(1):e1013357. doi: 10.1371/journal.pcbi.1013357 (PMC12810905; doi:10.1371/journal.pcbi.1013357)
Supplement: Attachment — Submitted filename: Review Response.pdf [file pcbi.1013357.s012.pdf]

Dear Editors,

We thank the reviewers for their comments and changed the manuscript accordingly, see below to our response to them.

Sincerely,

Steven X Wu, Chris N Davis, Mark Arnold, Mike J Tildesley

Reviewer #1: Thank you for addressing my comments. I appreciate the thoughtful revisions and the authors' efforts to respond to the majority of the points raised. However, several comments appear to be only partially addressed and could benefit from further clarification or expansion to fully meet their intent.

1. Previous comment: Add a brief overview of why HPAI H5N1 matters globally and why backyard flocks are a unique concern.

New comment: The authors have partially addressed this by providing examples from the UK and Southeast Asia that highlight the elevated risk in backyard flocks. While this supports the concern about their vulnerability, it does not clearly explain the global significance of HPAI H5N1, such as its impact on public health, poultry industries, or zoonotic potential.

Thank you for your comment, we have added the following to the first paragraph of the introduction. This now addresses the impact on public health and zoonotic potential of H5N1. We also highlighted in the introduction that backyard flocks can be a unique concern compared to commercial flocks.

"…These events are examples of HPAI H5N1 causing significant economic losses and disrupting food security. The rare but significant bird-to-human spillover cases shows its pandemic potential, which is further signified by the recent cases of mammalian infections. Backyard and smallholder flocks are a unique concern in the context of HPAI H5N1 epidemiology, since they are widespread globally, often have limited biosecurity compared to commercialised farms, and potentially have consistent surveillance measures. These factors make them a persistent reservoir of viral circulation and a risk for widespread transmissions and zoonotic exposure…"

2. Previous comment: Highlight novelty by emphasizing what makes your study different from existing research.

New comment: Thank you for partially addressing this point. To more clearly highlight the novelty of your study, I recommend explicitly stating early in the discussion how your approach differs from prior work. For instance, you could emphasize the unique focus on backyard mixed-species flocks, your assumptions around passive surveillance, or specific modeling choices that distinguish your study.

We thank the reviewer for the clarification. We added a sentence at the very start of the discussion, this explicitly states the novelty we believe this paper has:

"Our study differs from current literature with our unique focus on backyard, mixed-species flocks, the biological differences of each species, and the effectiveness of passive surveillance. To our knowledge, this is the first modelling study to quantify H5N1 transmission and passive surveillance performance in backyard mixed-species flocks, explicitly incorporating species-specific differences in infectiousness and mortality."

We also expanded on this in the second last paragraph of the introduction:

"… In this study, we aim to address the current gap in literature by focusing on backyard, mixed-species flocks and investigating the effectiveness of passive surveillance…"

3. Previous comment: Briefly outline how the findings could inform public health or agricultural policy.

New comment: Thank you for the additions that begin to connect your findings to policy relevance. To fully address the comment, I suggest summarizing how specific results, such as duck-related detection delays and species composition effects, could inform concrete public health or agricultural policies. A clear statement of practical implications would help readers understand how your findings can support decision-making.

We changed the second last paragraph of our discussion significantly to make the practical implications explicit, addressing the need to look at species-composition and use strategies accordingly based on our results. See the edited manuscript for exact changes.

4. Previous comment: The differences between duck-only and chicken-only flocks are interesting. To strengthen the interpretation, the authors should more clearly explain how biological traits such as longer infectious periods or lower mortality in ducks relate to the simulation results.

New comment: Thank you for adding a sentence to address this point. To strengthen the interpretation further, I suggest expanding the explanation of how these biological traits specifically influence simulation outcomes such as detection time, infection burden, and mortality patterns. This would help clarify the mechanisms driving the observed differences.

Some edits were made to the first paragraph of the discussion. Noting how the biological traits affect detection time and burden of infection.

"The simulation results match expectations, as ducks have a longer infectious period and lower mortality rate. These biological traits directly influence our simulation outcomes, as the prolonged infectious period allows ducks to infect other birds over an extended time frame, while the lower mortality means outbreaks can remain undetected for longer under a passive surveillance system that triggers on deaths. We hypothesise that ducks can act as a more persistent intermediate source of infection, which could lead to more widespread infections when a larger chicken population coexists with the ducks. We also found that ducks tend to contribute to a higher unnoticed burden of infection, again driven by the longer infectious period and lower mortality rate, which explains the higher cumulative infection burden, delayed detection, and increased potential for between-flock transmission and zoonotic exposure through direct or indirect contact between flocks and humans. It is important to note that our analysis focuses on detected outbreaks, which may underestimate the true burden of infection, particularly in duck-only flocks. The undetected outbreaks, while only occurring in duck flocks under certain parameter assumptions, could prolong viral circulation and increase the risk of transmission to nearby flocks."

5. Previous comment: The analysis of detection time at various death thresholds is valuable. To improve its practical relevance, it would help to discuss how these thresholds reflect real-world surveillance practices, particularly in smallholder and commercial settings.

New comment: Thank you for partially addressing this comment. To improve practical relevance, I suggest briefly discussing how the selected death thresholds align with actual surveillance practices in both smallholder and commercial contexts. Referencing typical reporting criteria or known behavioral patterns could help contextualize the thresholds and strengthen the policy implications.

Thank you for your input. We would like to note that the reporting criteria can be highly varied between farms and contexts, so it is difficult to say what is "typical". We added a reference to justify the use of mortality as a criterion.

"… Another investigation by Elbers et al. shows that irregular mortality rate is one of the main criteria for voluntary reporting in the Netherlands…"

We also further clarified our choice of thresholds:

"These death thresholds are intended to reflect likely farmer behaviour in small backyard flocks, where voluntary reporting is common, whereas for commercial flocks it is often viable to have regulatory surveillance and laboratory confirmation, which may detect infection at lower relative mortality or earlier clinical signs."

6. Previous comment: Using bird-days to measure infectious burden is appropriate. To highlight its epidemiological importance, the authors could more clearly explain how a higher burden might increase the risk of transmission to nearby flocks or humans.

New comment: Thank you for addressing this point. The added explanation strengthens the epidemiological relevance of bird-days. If applicable, consider briefly noting whether prolonged infectiousness in ducks could also elevate zoonotic risk to further clarify the public health implications.

This is addressed together with point 4 here:

"…and increased potential for between-flock transmission and zoonotic exposure through direct or indirect contact between flocks and humans."

7. Previous comment: The decision to analyze only simulations with detected outbreaks may underestimate the true burden of infection, particularly in duck-only flocks. A brief discussion or sensitivity analysis of undetected outbreaks would provide a more complete picture.

New comment: Thank you for including the bar plot in the supplementary materials. To fully address the comment, I suggest briefly discussing the implications of undetected outbreaks in the main text. For example, highlighting how these silent infections, especially in duck-only flocks, could influence surveillance strategies or underestimate transmission risk would reinforce the importance of your findings.

Again, addressed together with point 4:

"It is important to note that our analysis focuses on detected outbreaks, which may underestimate the true burden of infection, particularly in duck-only flocks. The undetected outbreaks, while only occurring in duck flocks under certain parameter assumptions, could prolong viral circulation and increase the risk of transmission to nearby flocks."

8. Previous comment: The authors acknowledge variability in farmer behavior, which is commendable. However, the discussion could be strengthened by referencing more region-specific behavioral studies or proposing how behavioral heterogeneity could be modeled in future work.

New comment: Thank you for citing relevant work on behavioral heterogeneity. To fully address the comment, I suggest referencing region-specific behavioral studies related to HPAI or smallholder poultry farming if available. Additionally, proposing how behavioral variability could be incorporated into future models, especially in the context of backyard flocks, would improve the relevance and depth of the discussion.

Thank you, we have added two studies that addressed some additional concerns with smallholder poultry farming in the discussion.

"Some farmers may also be hesitant to report if the signs of HPAI is not sufficiently clear [28], or try to rapidly harvest and sell birds during an outbreak [29]."

For the future work, a work has already been cited (Hill et al., 2023) to demonstrate the possibility of incorporating behavioural studies in mathematical models. We elaborated more on how to approach this work.

“For example, using surveys or interviews to group farmers by their knowledge level, reporting tendency, or response to flock mortality, as similarly demonstrated in Hill et al. [30].”

9. Previous comment: The discussion mentions important assumptions, such as homogeneous mixing and the exclusion of natural mortality. However, their impact on the results should be explored in more depth. For instance, how could spatial separation or age-related mortality affect detection patterns

New comment: Thank you for the update. To fully address the comment, I suggest briefly summarizing in the main text how key assumptions such as homogeneous mixing and exclusion of natural mortality might influence detection patterns. For example, spatial separation could slow transmission and delay detection, while age-related mortality might obscure outbreak signals. A clearer discussion of these mechanisms would help readers interpret the robustness of your findings.

We have fully rewritten the limitation paragraph based on another reviewer’s request. Homogeneous mixing and natural mortality are addressed there.

10. Previous comment: The author could consider presenting the balance between reducing mortality and limiting transmission as a multi-objective optimization problem to strengthen the study’s computational relevance.

New comment: Thank you for acknowledging the suggestion. To fully address the comment, I recommend briefly elaborating on how the trade-off between reducing mortality and limiting transmission could be framed as a multi-objective optimization problem. Even a short mention of potential objectives, constraints, or modeling approaches would help clarify the computational relevance and open avenues for future work.

We thank the reviewer for their suggestion and added this as the final sentence of the paper:

“For example, a multi-objective optimisation problem comparing the trade-off between reducing mortality and limiting transmission can be investigated in the context of multi-species flocks, which would enable a more systematic analysis of different surveillance strategies.”

Reviewer #2: The manuscript has been substantially improved. I do not see any remaining issues and therefore accept the manuscript.

Reviewer #3: The authors have addressed most of my previous comments well. However, several of the responses require further discussion or additional clarification.

First, I am not fully convinced by the following response: “We believe that density-dependent models are more appropriate for small flocks. Unlike in large-scale farms, where each bird is more likely to have a fixed number of contacts regardless of population increase, in a small backyard the population size directly impacts the contact rate of birds.”

The extent of density-dependent transmission is influenced not only by the number of animals but also by the space available to them. While the combined effects of these factors are complex to understand, I believe that in backyard settings, birds are likely to have sufficient space to roam and express their social behaviours more freely. This could lead to more heterogenous mixing and frequency-dependent transmission, as birds would not need to interact with unrelated individuals against their social preferences. I acknowledge that when bird density is very high relative to the available space, movement may be restricted, and contacts may occur mostly among nearby individuals (so may have a fixed number of contacts regardless population increase as the authors wrote). However, such settings are unlikely to represent typical backyard settings I imagine. One could also favour the opposite dynamics (if density is high but birds can still move, they would have more contacts with related birds, resulting in density-dependent transmission).

Could you please provide a further justification for your assumptions regarding transmission mode and include that in the manuscript?

We acknowledge that transmission mode is influenced by both flock size and available space. In typical UK backyard settings, birds are often confined to relatively small, fenced

enclosures rather than free-ranging across large open spaces (McClaughlin et al., 2024). Under such conditions, increasing flock size restricts movement and increases contact opportunities, regardless of species difference, supporting density-dependent transmission. In South East Asian countries, some smallholders keep their birds confined in enclosures in a similar manner to the UK, whilst others allow their birds to roam freely over a larger area. In the latter case, an assumption of frequency dependent transmission may more accurately capture transmission dynamics but for many smallholder farms, including premises in the UK and in South East Asia, we believe that density dependence is the appropriate assumption.

Overall, modelling backyard flocks can be highly-context dependent, and there are arguments to be made in both cases. Therefore, we are clear in our assumption of density-dependent transmission and include the following paragraph into the discussion:

"In our model, we assumed density-dependent transmission, such that the spread of disease through the flock depends on the total number of birds on the premises. In countries such as the UK, backyard poultry are typically kept in enclosures, and as flock size increases, the potential for contact also increases. However, in South East Asia, while some smallholders keep their poultry similarly enclosed, on other premises, birds are allowed to roam across much larger areas. In these cases, it may be more appropriate to assume frequency-dependent transmission, as birds are more likely to interact with a fixed number of contacts. The habitable space of the flock can also influence contact rates, so larger farms may have lower overall transmission rates than smaller farms. Furthermore, the inter-species transmission rate may be lower than assumed if chickens and ducks are kept separately, which violates our assumption of a homogeneously mixing population of birds. Some parameters, such as the probability of chickens showing clinical signs and dying from H5N1, were based on literature evidence and may vary in real-world settings. Additionally, our model had limited consideration of variance in farming conditions and environmental factors, which may impact its accuracy in specific backyard settings."

Second, please clarify why only layer chickens were chosen as the study population. Are layer chickens the most common type kept in backyard settings, and is this specific to the UK? I am not sure this assumption is generalisable, as in many Southeast Asian countries, for example, chickens are often raised primarily for meat production.

In the original review, the reviewer asked us to modify the manuscript to state that we were modelling commercial broiler premises. However, as this paper is focused upon smallholder backyard premises, we clarified that in the revised version. That said, we agree

with the reviewer that backyard premises can contain both layers and broilers and we have modified the methods of our manuscript to make this explicit. We do not use separate parameters for broilers and layers given the lack of available data to distinguish between the different bird types, but we note this limitation in the discussion section of our manuscript.

Finally, I remain unconvinced by the authors' rationale for excluding background mortality. Background mortality is a key factor in passive surveillance on poultry farms, and detection time is one of the main outcomes of this study. If ducks and chickens have different background mortality rates, this could influence detection times to some extent. Exploring this aspect would strengthen the manuscript, or alternatively, please provide a more robust justification for not including it.

Thank you for the comment. We agree that background mortality can influence detection dynamics. However, similar to our argument to frequency/density-dependent transmission, background mortality rate is highly varied across different farm management styles and regions, making any assumed rates speculative and context dependent. We have added two plots in the supplementary materials to show that for background mortality rate = 0.001 (approximately 30% yearly mortality without HPAI), the impact of background mortality is minimal and does not change the qualitative result. We acknowledge that including background mortality could be important for specific contexts. However, incorporating such effects would require better empirical characterisation of background mortality distributions in small mixed-species flocks, and we do not yet have the dataset for it. For this reason, we decided to present background mortality only in a supplementary sensitivity analysis rather than in the core model.

Reviewer #4: The authors have addressed my previous comments. I have no further comments.
