# Supplementary material for: The role of ducks in detecting Highly Pathogenic Avian Influenza in small-scale backyard poultry farms
Source: PLoS Comput Biol. 2026 Jan 9;22(1):e1013357. doi: 10.1371/journal.pcbi.1013357 (PMC12810905; doi:10.1371/journal.pcbi.1013357)
Supplement: Attachment — Submitted filename: PCOMPBIOL-D-25-01462 review comments.docx [file pcbi.1013357.s010.docx]

PCOMPBIOL-D-25-01462 Review Comments

Overall comment:

This study evaluates the implications of including ducks in mixed-species, small-scale backyard flocks for the transmission and detection of HPAI H5N1 virus. As ducks are relatively less susceptible to this virus, the findings are largely expected. Nevertheless, the authors present the epidemiological impacts in a systematic and informative way. I believe, however, that additional work is needed to be done to strengthen the manuscript for publication in this journal. My main comments relate to: (1) background mortality, (2) model assumptions, and (3) sensitivity analyses.

Specific comments:

Line 6: This example seems too specific, especially given that the journal targets an international readership. Nationwide H5N1 outbreaks have also occurred in other European and non-European countries.

Line 11: It may also be relevant to note that outbreaks were reported in cat shelters and mink farms, where close human-to-cat contact is common, for example, in both South Korea and Europe during comparable periods.

Line 44: It would be great to add contextual background on small-scale backyard flocks: indicate where these production systems are prevalent, and explain how this context affects the usefulness of your results.

Line 75: Given that you are considering ‘mixed species’ and ‘backyard’ flocks, how reasonable these assumptions are? On backyard farms, birds have relatively more freedom to move around compared to those on commercial farms, hence they may not interact homogeneously. Moreover, you have two different species in the same flock. I think these conditions might have some implications for the homogenous and density-dependent assumptions?

Line 96: I appreciate that the authors acknowledged the limitations of the available transmission parameters. One additional point I would like to raise is the heterogeneity that exists among chicken breeds, which may be particularly relevant given that the study population includes settings where indigenous breeds are commonly raised alongside ducks. For example, Sonali and Desi chickens exhibit different susceptibility to infection compared with commercial broiler breeds in Bangladesh where mixed-species flocks are common (e.g. https://wwwnc.cdc.gov/eid/article/24/12/18-0879_article). If the aim of the study is to assess the impact of birds with different infection courses, it may be helpful to clearly define “chickens” as commercial broiler breeds at the beginning of the manuscript. I believe commercial broiler chickens are what you meant based on my reading of the following paragraph.

Line 146: It would be helpful to define these thresholds as ‘background mortality’ and include them in Table 1, with appropriate references. In fact, I also wonder why background mortality was not incorporated directly into the model. Moreover, outbreak detection depends not only on background mortality but also on farmers’ sensitivity to observed deaths. Stochastic behaviour of these elements would impact detection, along with the role of ducks in your model? The current approach may oversimplify outbreak detection dynamics. I encourage the authors to incorporate these aspects into the model or, at minimum, discuss them in greater detail in the discussion.

Line 173: What would be the impact of background mortality and its difference between chickens and ducks (if present)? I would expect the effect to be negligible, given the relatively small flock sizes and short outbreak periods. However, it would have been helpful if the authors had addressed this in their model or at least showed that they had considered and justified this assumption.

Line 245: And there contact patterns might not be homogeneous…

Table 1: Please change Sensitivity and Derived into Assumed. You could explain different values of *k* and *p_d* values were tried as part of sensitivity analysis.

Line 134: What about conducting sensitivity analyses on transmission parameters derived from the literature? Given the limited number of studies, particularly those informing transmission rates, I think sensitivity analyses of these parameters are important. For instance, would the same trends hold under higher or lower transmission rates? Currently, sensitivity analyses were done assuming these parameter values are solid.
